# The Nonlinear Influence of Environmental Regulation on the Transformation and Upgrading of Industrial Structure

**DOI:** 10.3390/ijerph19148378

**Published:** 2022-07-08

**Authors:** Shuai Guan, Jinquan Liu, Yongfu Liu, Mingze Du

**Affiliations:** 1 School of Economics and Statistics, Guangzhou University, Guangzhou 510006, China; 339338@gzhu.edu.cn (S.G.); jinquan@gzhu.edu.cn (J.L.); 2 Business School, Jilin University, Changchun 130012, China; dumz18@mails.jlu.edu.cn

**Keywords:** environmental regulation, rationalization of industrial structure, upgrading the industrial structure

## Abstract

This paper measures the transformation and upgrading of industrial structure from two aspects of rationalization and upgrading of industrial structure, and empirically analyzes the impact of environmental regulation on industrial structure transformation and upgrading by using data of 29 provinces in China from 2004 to 2015. It was found that there is a significant nonlinear effect between environmental regulation and the transformation and upgrading of industrial structure. Specifically, environmental regulation is not conducive to the rational development of industrial structure, but with the continuous improvement of economic development level and human capital level, the inhibitory effect of environmental regulation on the rationalization of industrial structure is gradually weakened. The influence coefficient of environmental regulation on the rationalization of industrial structure is 0.0619~0.2648. Moreover, environmental regulation effectively drives the upgrading of industrial structure, and when the level of economic development and human capital are higher than the threshold, the role of environmental regulation in promoting the high development of industrial structure is gradually enhanced. The influence coefficient of environmental regulation on the upgrading of industrial structure is 0.0540~0.5626. Therefore, it is of great significance to formulate appropriate environmental regulation policies according to local conditions in the transformation and upgrading of industrial structure.

## 1. Introduction

Continuous economic restructuring, especially industrial restructuring, is an important source of economic growth and a necessary prerequisite for maintaining high-quality economic development. At this stage, China’s economic development has entered a new era, and the basic feature is that the economy has changed from a high-speed growth stage to a high-quality development stage. Relying on the traditional extensive development mode, high environmental pollution and ecological damage have hindered the process of economic structure transformation. Environmental governance has become an unavoidable top priority in China’s transformation of development mode and optimization of economic structure. Since the 1980s, Chinese governments at all levels have gradually established and formulated relatively perfect environmental protection systems and policies to reduce pollution emissions and improve environmental quality, and achieved certain results. However, the unsustainable development mode of exchanging environmental pollution for economic growth for a long time has led to the serious situation of environmental pollution in various regions.

The transformation and upgrading of industrial structure based on breaking the constraints of energy and resources and alleviating the pressure on the ecological environment is an important way to achieve a win-win situation between environmental protection and economic growth. It is an important starting point for China to realize the green economy and sustainable development to promote the transformation of industrial development mode from traditional factor driven to intensive innovation driven and from low value-added to high value-added through the pressure of environmental constraints brought by environmental regulation. The transformation and upgrading of industrial structure is the core tool to coordinate the economy and environment. On the one hand, the industrial structure is directly related to how the economic system uses resources and discharges waste. Industrial structure is not only the converter of natural resource input, but also the control body of the quantity and type of pollutants. On the other hand, environmental regulation will increase the cost of enterprises. Driven by maximizing profits, enterprises adjust production behavior and cause changes in industrial structure. Therefore, it is necessary for us to organically combine the transformation and upgrading of industrial structure with research on environmental protection. As a necessary means for the government to protect the environment, studying its impact on the transformation and upgrading of industrial structure has theoretical and practical significance for realizing environmental protection and structural transformation.

In fact, the deteriorating ecological environment has not allowed China to wait for the unknown inflection point in the environmental Kuznets curve, and appropriate intervention is needed to achieve green economic development [1]. As a necessary means for the government to protect the environment, whether environmental regulation can go hand in hand with industrial transformation and upgrading is worth further study. The representative results of the early academic research on the economic effects of environmental regulation are the “compliance cost” and “Porter hypothesis”. “Compliance cost” starts from a static perspective and assumes that the technological level, resource allocation, production process and consumer demand of the enterprise remain unchanged. It assumes that strict environmental regulations increase additional cost of pollution control for enterprises, so as to make the enterprise production ability and profit levels drop, weaken enterprise competitiveness, and ultimately hinder economic growth. Based on a dynamic perspective, the “Porter Hypothesis” holds that appropriate environmental regulation can motivate innovative activities and optimize resource allocation in order to reduce costs, stimulate the “innovation compensation” effect, and then promote the improvement of production efficiency and the enhancement of competitive strength, so that environmental protection and economic growth can be balanced.

In recent years, many scholars have empirically tested the Porter hypothesis according to different hypotheses and obtained different conclusions. Most of the findings support the Porter hypothesis. For example, some scholars found that the increase in enterprises’ pollution emission reduction expenditures can promote the growth of environmental protection patent applications, and this relationship is very prominent in industries with strong international competitiveness, which supports the weak Porter Hypothesis [2,3]. Some studies distinguish between regulation-induced and voluntary environmental innovations. Both regulation-induced and voluntary innovation can improve enterprise resource efficiency and profitability, but regulation-induced innovation has a greater impact. However, the Porter hypothesis does not hold in general for its “strong” version, but depends on the type of environmental innovation [4]. In addition, some scholars have also proved with the strong Porter hypothesis that stricter environmental policies improve growth and the environment and induce profitable innovation [5]. Moreover, some studies used Chinese panel data to conduct empirical tests and found that higher environmental regulation intensity could promote technological progress and green total factor productivity, which supports the Porter hypothesis [6,7,8,9].

Other studies have rejected the Porter hypothesis. Jaffe and Palmer (1997) [10] tested the Porter hypothesis by using panel data of the US manufacturing industry and pointed out that although the cost of environmental regulation could increase R&D expenditure, it had no impact on innovation output. They also pointed out that some supporters of “Porter hypothesis” used case studies that were not rigorous enough and did not provide a basic criterion for reasonable environmental regulation. Ederington and Minier (2003) [11] found that strict environmental regulation had a great impact on net imports, that is, environmental regulation weakened the competitiveness of enterprises. Some studies have analyzed the relationship between environmental regulation and ecological innovation, and found that only long-term goals and market incentives are positively correlated with ecological innovation. Traditional regulatory tools, namely legally binding tools, cannot effectively trigger innovation behavior at the enterprise level. Refs. [12,13] used mixed regression and systematic GMM methods to study the impact of different environmental regulation tools on China’s energy conservation and emission reduction technologies, and the results did not support the weak Porter hypothesis.

From the current research progress, scholars mainly focus on the relationship between human capital [14,15,16,17], trade opening [18,19,20], financial development [21,22,23,24], industrial policy [25,26] and the change in industrial structure. The discussion on the impact of environmental regulation on industrial transformation and upgrading has gradually begun to grow, and many scholars have drawn different conclusions based on different empirical methods. Xiao and Li (2013) [27] found that environmental regulation mainly affects the transformation and upgrading of industrial structure through technological innovation, demand and international trade. Moreover, environmental regulation plays a positive role in promoting the direction and path of industrial upgrading, and environmental regulation and industrial structure upgrading can achieve a win-win situation. Guo and Yuan (2020) [28] studied the forcing effect of environmental regulations and government R&D subsidies on the upgrading of industrial structure, and found that the coupling effect of environmental regulations and government R&D subsidies significantly enhanced the “innovation compensation” effect, which was conducive to promoting the upgrading of industrial structure. Some scholars have investigated the impact of informal environmental regulation on the upgrading of industrial structure. As an external binding force, informal environmental regulation in the form of environmental media reports promotes the upgrading of industrial structure by increasing the dual pressure of local government supervision and public opinion [29].

However, some scholars have come to different conclusions. Zhong et al. (2015) [30] theoretically analyzed the impact of environmental regulation on corporate behavior, and then verified the relationship between environmental regulation and industrial structure adjustment by using the panel threshold model. The study showed that there was a U-shaped curve relationship between the two, and only when the threshold value was crossed, environmental regulation could effectively force industrial structure transformation and upgrading. Shen et al. (2020) [31] drew a similar conclusion, namely, that only a higher intensity of environmental regulation could effectively promote the transformation and upgrading of the manufacturing industry. Moreover, some studies show that when the level of economic development is low, the impact of environmental regulation on industrial structure upgrading is not significant. Only when the level of economic development tends to be high, environmental regulation will significantly promote green technology innovation and industrial structure upgrading [32].

The purpose of this paper is to examine the nonlinear impact of environmental regulation on industrial structure transformation and upgrading. In the following part of this paper, the PSTR model is used to test the relationship between environmental regulation and industrial upgrading on the basis of controlling relevant variables, and the robustness test is carried out. The analysis of the relationship between environmental regulation and industrial structure transformation and upgrading can provide reference for the government to make selective environmental regulation decisions. Compared with previous studies, the innovation of this paper is mainly reflected in the following three aspects: (1) In terms of ideas, this paper further considers the impact of environmental regulation on the transformation and upgrading of China’s industrial structure when there are differences in the level of economic development and human capital, and expands and supplements previous studies on single factors. (2) Methodologically, in contrast with previous regression equation analysis and in order to verify the continuous and gradually changing nonlinear effect of environmental regulation on the transformation and upgrading of industrial structure, this paper uses the panel smooth transition regression (PSTR) model proposed by González et al. (2004) [33]. A series of estimates and tests are carried out for smooth transformation effects and parameters of functions with exogenous variables, so as to reflect the nonlinear characteristics of the problem analyzed and the gradual behavior of transformation. (3) In terms of data, this paper constructs a more basic and comprehensive index of environmental regulation, so as to reflect the intensity of environmental regulation in China. In addition, this paper measures the transformation and upgrading of industrial structure from the two aspects of rationalization and upgrading of industrial structure. Compared with the previous industrial structure upgrading index measurement, this method can more objectively reflect the upgrading level of China’s regional industrial structure.

## 2. Methods

### 2.1. The PSTR Model

This paper uses the panel smooth transition regression model (PSTR) proposed by González et al. (2004) [33], which avoids the problem of heterogeneity in a nonlinear modeling specification. The two-regimes form of the PSTR model is as follows:

(1) $$y_{it}=\mu_{i}+\beta_{0}^{\prime }x_{it}+\beta_{1}^{\prime }x_{it}g(q_{it};\gamma ,c)+u_{it}\;\;\;i=1,2,\cdots ,N,\;\;\;t=1,2,\cdots ,T$$

where $y_{it}$ is dependent variable, $x_{it}$ is vector of explanatory variables, $\mu_{i}$ is the fixed effect, $u_{it}$ is error term. $g(q_{it};\gamma ,c)$ is transition function, and its value ranges from 0 to 1. γ>0 is smooth parameter and c outlines the threshold candidate variable. The logical transition function is defined as follows:

(2) $$g(q_{it;}\gamma ,c)={\left\{1+\exp\left[-\gamma \overset{m}{\underset{j=1}{\Pi }}(q_{it}-c_{j})\right]\right\}}^{-1}\lambda >0,c_{1}\leq c_{2}\leq \cdots \leq c_{m}$$

In logical transition function, $c=(c_{1},\cdots c_{m}{)}^{\prime }$ represents an *m*-dimensional vector containing the location parameters, and γ > 0 and $c_{1}\leq c_{2}\leq \cdots \leq c_{m}$, $c_{m}$ describes the identification restrictions. Actually, the PSTR model with multiple regimes is specified as follows:

(3) $$y_{it}=\mu_{i}+\beta_{0}^{\prime }x_{it}+\sum_{j=1}^{r}\beta_{j}^{\prime }x_{it}g_{j}(q_{it}^{j};\gamma_{j},c_{j})+u_{it}$$

where the transition function $g_{j}(q_{it}^{j};\gamma ,c_{j})$, j=1,⋯r, depends on the slope parameters $\gamma_{j}$ and location parameters $c_{j}$. If $\gamma_{j}\to \infty$, the model in Equation (3) will be a panel threshold regression (PTR) model; if $\gamma_{j}\to 0$, then the model in Equation (3) will be a linear panel regression model.

Before estimating the model, it is necessary to test the linearity of the model, that is, to test whether $\gamma_{j}$ in Equation (3) is equal to 0. However, under that assumption, the model contains unknown redundant parameters. One of the solutions is to perform first-order Taylor expansion on the transition functions at $\gamma_{j}=0$, and the auxiliary regression is rewritten as follows:

(4) $$y_{it}=\mu_{i}+\beta_{0}{{}^{\prime }}^{*}x_{it}+\beta_{1}{{}^{\prime }}^{*}x_{it}q_{it}+\cdots +\beta_{m}{{}^{\prime }}^{*}x_{it}q_{it}{}^{m}+u_{it}$$

Therefore, the linear test of the PSTR model is changed to test $H_{0}:\beta_{1}{}^{*}=\cdots =\beta_{m}{}^{*}=0$. Under this assumption, $SSR_{0}$ and $SSR_{1}$ are defined as the sums of the square residuals of the linear fixed effect model and the two-regimes PSTR model, respectively. In this study, we employed the three tests as follows.

(5) $$LM_{\chi^{2}}=TN\frac{\left(SSR_{0}-SSR_{1}\right)}{SSR_{0}}$$

(6) $$LM_{F}=\frac{\left(SSR_{0}-SSR_{1}\right)}{mk}/\frac{SSR_{0}}{\left(TN-N-m(k+1\right)}$$

(7) $$LRT=-2\left[\log(SSR_{0})-\log(SSR_{1})\right]$$

In the above test statistics, *T* is the time length of panel data, *N* is the number of individuals, *m* is the number of location parameters in the transfer function, and *k* is the number of explanatory variables. In addition, under the null hypothesis, the $LM_{\chi^{2}}$ statistic is distributed as $\chi^{2}\left(mk\right)$, the $LM_{F}$ statistic has an approximate $F\left(mk,TN-N-mk\right)$ distribution, and the LRT statistic has also $\chi^{2}\left(mk\right)$ distribution.

### 2.2. Variable Selection and Processing

#### 2.2.1. Explanatory Variable

The explanatory variable in this paper is the level of industrial structure adjustment, which is mainly measured from two dimensions: rationalization of industrial structure (RIS) and upgrading the industrial structure (UIS). The rationalization of industrial structure not only reflects the ability of structural transformation between industries, but also reflects the degree of effective utilization of resources, which is a measure of the coordination degree of factor input and output structure [34]. In terms of this degree of coordination, researchers generally use the degree of structural deviation to measure the rationalization of industrial structure, but this indicator treats the economic status of the three industries as equal, ignoring the importance of different industries in the economy [35]. This paper selects the Theil index to measure the rationalization level of industrial structure in each region, which measures the deviation of output value and employment structure of each industry as well as the difference in economic status of each industry [36]. The specific calculation formula is as follows:

(8) $$TL=\sum_{m=1}^{3}\left(\frac{Y_{m}}{Y}\right)\ln\left(\frac{Y_{m}}{L_{m}}/\frac{Y}{L}\right),m=1,2,3$$

where *TL* is Theil index, *Y* is output, *L* is employment, and *m* is the three major industries. The Theil index can better reflect the output value structure and employment structure of China’s three major industries. When TL=0, the economy was in a balanced state, and the larger the Theil index was, the more likely the economic development was to deviate from the balanced state, and the industrial structure was unreasonable.

The upgrading of industrial structure is an important part of the upgrading of industrial structure, which reflects the dynamic evolution process of industrial structure from a low level to high level under different economic development. According to Clark’s law, the elevation of industrial structure is defined as the increase in the proportion of non-agricultural output value in the general literature. However, this traditional measurement method, which only focuses on the increase in industrial share, cannot accurately reflect the nature of industrial structure evolution. The upgrading of industrial structure involves the evolution of the proportional relationship between industries and the improvement in labor productivity. If industries with higher labor productivity occupy a larger share in an economy, it indicates that the industrial structure of the region is at a higher level of sophistication [37]. This paper defines the advanced industrial structure as the product of the proportional relationship between industries and industrial labor productivity, and the specific calculation formula is as follows:

(9) $$ES=\sum_{m=1}^{3}\frac{Y_{m}}{Y}\times LP_{m},m=1,2,3$$

where *ES* is upgrading of industrial structure, *LP* is labor productivity, which is obtained by using the ratio of regional industrial added value to the employed personnel at the end of the same period. If the value is rising, it means that the industrial structure is upgrading.

#### 2.2.2. Core Explanatory Variable

When measuring the intensity of environmental regulations, many methods are often used in the earlier literature. Based on the comprehensive index method proposed by Fu (2010) [38], the environmental regulation index is constructed, and the four individual indexes of sulfur dioxide removal rate, industrial smoke and dust removal rate, comprehensive utilization rate of industrial solid waste and harmless treatment rate of domestic waste are weighed to obtain the environmental regulation benefit index. The calculation process is as follows: First, all kinds of indicators are linearly standardized (and the calculation formula is shown in Equation (10)). Secondly, the weight value of each index is calculated (the calculation formula is shown in Equation (11)). Finally, the standardized values and weights of various indicators are used to calculate environmental regulation income indicators. See Equation (12) for the specific calculation process.

(10) $$UE_{ij}^{s}=\left[UE_{ij}-MIN\left(UE_{j}\right)\right]/\left[MAX\left(UE_{j}\right)-MIN\left(UE_{j}\right)\right]$$

(11) $$W_{j}=\frac{E_{j}}{\sum E_{j}}/\frac{Y_{i}}{\sum Y_{i}}=\frac{E_{j}}{Y_{i}}\times \frac{\sum Y_{i}}{\sum E_{j}}=\frac{E_{j}}{Y_{i}}/\frac{\sum E_{j}}{\sum Y_{i}}=UE_{ij}/\bar{UE_{j}}$$

(12) $$ER_{i}=\frac{1}{4}\sum_{j=1}^{4}W_{j}\times UE_{ij}^{s}$$

where $UE_{ij}$ is the original value of indicators, $MIN\left(UE_{j}\right)$ and $MAX\left(UE_{j}\right)$ are the minimum and maximum values, respectively, of various pollution indicators in all regions each year, $UE_{ij}^{s}$ represents the standardized values of various indicators, $W_{j}$ is the adjustment coefficient of pollutants *j*, $E_{j}$ is the emission of pollutants, and $ER_{i}$ refers to the core explanatory variable. 

#### 2.2.3. Control Variable

Referring to the work of Zhang et al. (2010) and Jin et al. (2018) [39,40], three control variables are set in this paper.

*Urban* is the level of urbanization. This variable is measured by the ratio of urban population to total population at the end of the year. On the one hand, urbanization can significantly promote the transformation and upgrading of industrial structure. Urbanization promotes specialization and modern industrial agglomeration, promotes technological innovation, and provides impetus for industrial upgrading. Urbanization will also eliminate some extensive enterprises, while intensive and high value-added enterprises will be screened and retained, so as to optimize the industrial structure. On the other hand, a few scholars believe that the development of urbanization is not conducive to the optimization of industrial structure. The main reason is that the current international division of labor system can easily lead developing countries into a locked state at the bottom of the global value chain. Blind urbanization and energy-intensive industries together lead economic development along an uncharted path. Therefore, the development of urbanization is not conducive to the transformation and upgrading of industrial structure.

*Open* is the level of opening up. This variable is measured by the ratio of foreign direct investment stock to regional GDP. On the one hand, for developing countries, opening up is conducive to attracting foreign investment and promoting the development of emerging industries. Opening up promotes the integration of the host country into the global industrial division, promotes the rapid development of manufacturing, and then promotes the industrialization process. On the other hand, although opening to the outside world can quickly promote industrial development, it may not be conducive to the upgrading of industrial structure in the long run because the host country is mostly at the bottom of the global industrial chain, and it also brings serious environmental pollution and inhibits the improvement of labor productivity.

*Edu* is the level of human capital. This paper measures this variable based on the number of years of education per person over the age of 6. The improvement of human capital level is conducive to the improvement of enterprise technology level, and then the improvement of regional industrial structure. The higher the level of human capital, the higher the population density of highly educated workers, which is convenient for knowledge sharing to produce new ideas and fully meet the need for professional talent for the adjustment of industrial structure in the region. For regions with low levels of human capital, the serious outflow of talent prevents the satisfaction of demand for high-level human capital for industrial structure optimization, which leads to insufficient innovation power and weakens industrial structure adjustment. Therefore, human capital has become one of the main factors restricting the upgrading of industrial structure.

### 2.3. Model Setting

In this paper, we use the PSTR approach based on the two extreme regimes to test the nonlinear relationship between environmental regulation and RIS:

(13) $$\begin{array}{l} TL_{it}=\mu_{i}+\beta_{01}ER_{it}+\beta_{02}Urban_{it}+\beta_{03}Open_{it}+\beta_{04}Edu_{it}\\ +\left(\beta_{11}ER_{it}+\beta_{12}Urban_{it}+\beta_{13}Open_{it}+\beta_{14}Edu_{it}\right)g\left(Pgdp_{it};\gamma ,c\right)+u_{it} \end{array}$$

(14) $$\begin{array}{l} TL_{it}=\mu_{i}+\beta_{01}ER_{it}+\beta_{02}Urban_{it}+\beta_{03}Open_{it}+\beta_{04}Edu_{it}\\ +\left(\beta_{11}ER_{it}+\beta_{12}Urban_{it}+\beta_{13}Open_{it}+\beta_{14}Edu_{it}\right)g\left(Edu_{it};\gamma ,c\right)+u_{it} \end{array}$$

where economic development level is the transformation variable in Equation (13), and human capital level is the transformation variable in Equation (14). The elasticity of environmental regulation with respect to the RIS for the *i*th city at period *t* is given by:

(15) $$e_{it}=\beta_{01}+\beta_{11}g\left(q_{it};\gamma ,c\right)$$

As well, this paper uses the following PSTR approach based on the two extreme regimes to test the nonlinear relationship between environmental regulation and UIS:

(16) $$\begin{array}{l} ES_{it}=\mu_{i}+\beta_{01}ER_{it}+\beta_{02}Urban_{it}+\beta_{03}Open_{it}+\beta_{04}Edu_{it}\\ +\left(\beta_{11}ER_{it}+\beta_{12}Urban_{it}+\beta_{13}Open_{it}+\beta_{14}Edu_{it}\right)g\left(Pgdp_{it};\gamma ,c\right)+u_{it} \end{array}$$

(17) $$\begin{array}{l} ES_{it}=\mu_{i}+\beta_{01}ER_{it}+\beta_{02}Urban_{it}+\beta_{03}Open_{it}+\beta_{04}Edu_{it}\\ +\left(\beta_{11}ER_{it}+\beta_{12}Urban_{it}+\beta_{13}Open_{it}+\beta_{14}Edu_{it}\right)g\left(Edu_{it};\gamma ,c\right)+u_{it} \end{array}$$

where, similarly to above, economic development level is the transformation variable in Equation (16), and human capital level is the transformation variable in Equation (17).

## 3. The Nonlinear Relationship between Environmental Regulation and RIS

### 3.1. Data

Our empirical background uses annual data of 29 provinces and cities in China. The period of study is from 2004 to 2015. The data are obtained from the “China Environmental Yearbook”, “China Industrial Statistical Yearbook”, “China Economic Network Statistics Database” and “China Statistical Yearbook”. The sample and the period choice were constrained by the availability of data. Table 1 displays the descriptive statistics of the used variables.

### 3.2. Discussion of the Linearity Results

Table 2 and Table 3 respectively show the linearity hypotheses and no remaining nonlinearity test results of the PSTR model under different location parameter dimensions when treating economic development level and human capital level as transition variables. It can be seen from the results in Table 2 and Table 3 that in the case of m = 1 and m = 2, $LM_{\chi^{2}}$, $LM_{F}$ and LRT statistics reject the original hypothesis $H_{0}:r=0$ at the significance level of 5%. This means that there is a nonlinear relationship between environmental regulation and RIS. Further, no remaining non-linearity test of the PSTR model shows that the original assumption $H_{0}:r=1$ cannot be rejected when *m* = 1 or *m* = 2. It shows that the PSTR model only contains a nonlinear transition function, that is, *r* = 1. Next, we use AIC and BIC criteria to determine the best value of m. When *m* = 1, the AIC value and BIC value corresponding to the transition variable are less than the value when *m* = 2. Based on this, it can be concluded that the best combination of the number of transition functions and the dimension of position parameters in the model is *r* = 1, *m* = 1.

### 3.3. Discussion of the Empirical Results

The findings of the PSTR regression model are recorded in Table 4. Among these, columns (1) and (2) are the estimated results when economic development level and human capital level are used as transformation variables, respectively. According to the results in Table 4, the interpretation is as follows:

From column (1), we can see that the environmental regulation exerts a significant negative impact on RIS for the low and high regimes. The coefficient of environmental regulation on RIS is 0.2648 in the low regime and 0.0594 (0.2648–0.2054) in the high regime. It can be seen that with the gradual improvement of the level of economic development, the inhibitory effect of environmental regulation on the rationalization of industrial structure is gradually decreasing.

Specifically, the level of industrial structure in economically backward areas is low, and the coordination between industries is poor. At this time, the improvement of environmental regulation may aggravate the distortion of the factor market, affect industrial productivity, reduce the efficiency of resource allocation and hinder the rational development of industrial structure. With the gradual development of the economic level, the Party Central Committee began to implement the strategy of promoting regional coordinated development in order to reduce the regional gaps; each region combined with its own comparative advantages and industrial foundation were expected to pursue a reasonable layout of industrial structure, and the regional industrial transfer was promoted in an orderly manner. However, there is a large gap in the level of industrial structure between the east, central and western regions of China, and the problem of repeated industrial construction is still prominent. Under this background, the implementation of environmental regulation policy is not conducive to strengthening the correlation between industries and reduces the efficiency of resource allocation, resulting in the failure to give full play to the role of environmental regulation in promoting the rationalization of industrial structure.

From columns (2), it can be seen that the impact of environmental regulation on RIS is still significantly negative in both regimes. In the first regime, the coefficient of environmental regulation on RIS is 0.2382, and in the high regime, it is 0.0619 (0.2382–0.1763). It can be seen that with the improvement of human capital level, the negative effect of environmental regulation on the RIS is becoming smaller and smaller. The possible reason is that the improvement of the level of human capital will lead to the agglomeration of other factors of production (mainly material capital), which gives the industrial sectors and regions with high stock of human capital a comparative advantage in gathering resources. The agglomeration effect promotes the transfer and allocation of other production factors among industries and improves the rationality of industrial structure.

Furthermore, the urbanization level and opening up to trade have a significant effect on RIS. In the low regime, the urbanization level and opening up variables have a positive significant impact on RIS. However, in the high regime, they hinder the RIS. Moreover, human capital has a significant negative impact on RIS in the low regime. Nonetheless, it exerts a positive influence on RIS in the high regime.

### 3.4. Results of Robustness Test

In order to further test the robustness of the nonlinear relationship between environmental regulation and RIS, this paper uses the ratio of pollutant discharge fee income to industrial added value as an alternative variable for robustness analysis. The nonlinear test results of the model are shown in Table 5 and Table 6.

According to the results in Table 5 and Table 6, the relationship between environmental regulation and RIS is nonlinear. Next, the nonlinear least square method is used to estimate the model, and the estimation results are shown in Table 7. The estimated coefficient of environmental regulation is significant. When treating the level of economic development and the level of human capital as the transformation variables, the impact of environmental regulation on the RIS has nonlinear characteristics. With the improvement of economic level and human capital level, the negative impact of environmental regulation on RIS is gradually reduced.

To sum up, the estimation results using the ratio of pollutant discharge fee income to industrial added value as an alternative variable are robust. Compared with the above estimation results, the estimation coefficient symbols of explanatory variables are basically the same, and the significance level of variables does not change significantly, indicating that the estimation results of the above model are reliable.

## 4. The Nonlinear Relationship between Environmental Regulation and UIS

### 4.1. Discussion of the Linearity Results

The results of the linearity test and no remaining nonlinearity test are presented in Table 8 and Table 9. Whether economic development level or human capital level is used as a transformation variable, it is conclusive that the linearity hypothesis is rejected. The results achieved based on $LM_{\chi^{2}}$, $LM_{F}$ and LRT statistics also reflect the rejection of the null hypothesis, implying that the relationship between environmental regulation and UIS is nonlinear. Further, the no remaining non-linearity test of the PSTR model shows that the original assumption $H_{0}:r=1$ cannot be rejected when *m* = 1 or *m* = 2. It shows that the PSTR model only contains a nonlinear transition function, that is, *r* = 1. Next, we use AIC and BIC criteria to determine the best value of m. When *m* = 1, the AIC value and BIC value corresponding to the transition variable are less than the value when *m* = 2. Based on this, it can be concluded that the best combination of the number of transition functions and the dimension of position parameters in the model is *r* = 1, *m* = 1.

### 4.2. Discussion of the Empirical Results

The estimation results of the PSTR regression model are shown in Table 10. Among these, columns (1) and (2) are the estimated results when economic development level and human capital level are used as transformation variables, respectively. As shown in the table, the interpretation is as follows:

From column (1), we can see that environmental regulation exerts a significant positive impact on UIS for the low and high regimes. The coefficient of environmental regulation on UIS is 0.0859 in the low regime and 0.5626 (0.0859 + 0.4767) in the high regime. It can be found that with the gradual improvement of the level of economic development, the role of environmental regulation in promoting the UIS is also gradually increasing. The possible explanation for this is that with the improvement of the level of economic development, people’s demand for green and environmentally friendly products becomes stronger and stronger, and the pollution control costs for pollution-intensive industries increase, forcing them to carry out industrial transfer or increase investment in green technology innovation and develop environmentally friendly products. Environmental regulation drives the UIS by triggering an innovation compensation effect.

From column (2), it can be seen the impact of environmental regulation on UIS is still significantly positive in both regimes. In the first regime, the coefficient of environmental regulation on UIS is 0.0540, and in the second regime, it is 0.4747 (0.0540 + 0.4207). On both sides of the threshold, there are significant differences in the role of environmental regulation in promoting UIS, indicating that there is a nonlinear relationship between environmental regulation and UIS. It can be seen that with the improvement of human capital level, the role of environmental regulation in promoting UIS is gradually strengthened. As the carrier of technological progress, human capital is an important factor to promote industrial upgrading. In areas where human capital is at a high level, the improvement of environmental regulation can force enterprises to carry out technological innovation and promote the high development of industry by implementing innovation drives.

Furthermore, in both regimes, the urbanization level has a significant influence on UIS. Additionally, opening up can promote UIS in the low regime, but it does not promote UIS when the model is in the high regime. Moreover, whereas human capital has a significant negative impact on UIS in the low regime, it exerts a positive influence on UIS in the high regime.

### 4.3. Results of Robustness Test

In order to verify the robustness of the nonlinear impact of the environmental regulation on UIS, the ratio of pollutant discharge fee income to industrial added value in each region is further used as a substitute to regress the model again. The test results of model nonlinearity are shown in Table 11 and Table 12.

According to the results in Table 11 and Table 12, the relationship between environmental regulation and UIS is nonlinear. As shown in Table 13, when treating the level of economic development as the conversion variable, the impact of environmental regulation on the upgrading of industrial structure has significant nonlinear characteristics. With the improvement of regional economic development level, the industrial structure upgrading effect of environmental regulation is gradually relaxed. Environmental regulation promotes the high development of industrial structure by improving the level of green technology. When treating the level of human capital as the conversion variable, there is a significant difference in the impact of environmental regulation on the upgrading of industrial structure on both sides of the location parameter. With the continuous improvement of human capital level, the role of environmental regulation in promoting the UIS is gradually strengthened, and environmental regulation significantly drives the upgrading of industrial structure.

## 5. Discussion

### 5.1. Impacts of Environmental Regulation on RIS

The impact of environmental regulation on RIS has obvious nonlinear characteristics. Environmental regulation has not effectively promoted RIS, but with the improvement of economic development level and human capital level, the negative effect of environmental regulation on RIS has gradually weakened. The reason behind this is that local governments attract mobile resources such as capital and labor by relaxing environmental regulations, which hinders the free flow of capital and increases the replacement cost of factors, and is ultimately not conducive to the optimal allocation of resources among industries and the rational development of industrial structure. However, with the improvement of economic development and human capital, people’s requirements for environmental quality continue to improve. High-level environmental regulation promotes quality improvement at the macro level by promoting industrial transfer and internal structure optimization, which is conducive to industrial division and cooperation and the rational distribution of industrial space.

### 5.2. Impacts of Environmental Regulation on UIS

The impact of environmental regulation on UIS has obvious nonlinear characteristics. Environmental regulation effectively drives UIS. When the level of economic development and human capital continue to improve, the role of environmental regulation in promoting UIS is gradually enhanced. According to the Porter hypothesis, the appropriate intensity of environmental regulation can stimulate the innovation behavior of enterprises, and the resulting innovation compensation effect offsets the compliance cost caused by environmental protection and improves the competitiveness and competitive advantage of enterprises. Driven by technological progress, production factors and resources are transferred from low-productivity sectors to high-productivity sectors. High-productivity sectors gain development opportunities, and low-productivity sectors are gradually eliminated from the market, ultimately driving the optimization and upgrading of industrial structure. At the same time, with the continuous improvement of the level of economic development and human capital, the public’s awareness of environmental protection and requirements for environmental quality have increased, which has promoted the upgrading of industrial structure from the demand side. The tighter environmental regulation promotes the high-level development of the industry from the demand side by fine-tuning the enterprises, changing the production mode of enterprises and adjusting the product structure to respond to the changes in market demand.

### 5.3. The Overall Impact of Environmental Regulation

The above results show that environmental regulation does not promote RIS, but effectively promotes UIS. In fact, the impact of environmental regulation implementation on the transformation and upgrading of industrial structure is complex. Specifically, this paper applies the ratio of regional pollutant discharge fee income to industrial added value as an alternative variable of environmental regulation. Robustness analysis showed that the above research results are valid. In general, environmental regulation has not significantly promoted the transformation and upgrading of industrial structure, which has brought severe challenges to China’s industrial structure adjustment and high-quality economic development.

## 6. Conclusions

As a policy tool of local government, environmental regulation has an impact on the transformation and upgrading of industrial structure mainly through the rationalization of industrial structure (RIS) and upgrading of industrial structure (UIS). Environmental regulation effectively drives the transformation and upgrading of industrial structure only when it promotes RIS and UIS at the same time. Based on the panel data of 29 provinces in China from 2004 to 2015, this paper empirically tested the impact of environmental regulation on the rationalization and upgrading of industrial structure by using the PSTR model and treating the level of economic development and human capital as transition variables. Our study shows that environmental regulation has positive effects on UIS, but negative effects on RIS. In general, environmental regulation does not drive the transformation and upgrading of industrial structure.

This paper also has some shortcomings, which need to be further standardized in future research. We tested the nonlinear relationship between environmental regulation and the transformation and upgrading of industrial structure, but only provided a hypothesis regarding the possible reasons for the results, which will require further empirical testing of the theoretical mechanism of the impact of environmental regulation on the transformation and upgrading of industrial structure.

## Figures and Tables

**Table 1:** The descriptive statistics.

Variable	Mean	Std. Dev	Min	Max
TL	0.2484	0.1534	0.0161	0.8771
ES	0.7660	0.5090	0.0939	2.8188
ER	0.7518	0.4342	0.1277	2.7885
Pgdp	9.9567	0.5686	8.3703	11.1634
Urban	0.3948	0.1725	0.1576	0.9032
Open	1.9793	2.1058	0.2478	14.0070
Edu	8.6315	0.9672	6.3778	12.0807

**Table 2:** The linearity test and no remaining non-linearity test (transformation variable: *Pgdp*).

Hypotheses	m=1			m=2		
	$LM_{\chi^{2}}$	$LM_{F}$	LRT	$LM_{\chi^{2}}$	$LM_{F}$	LRT
linearity	9.859	2.296	10.001	46.149	5.943	49.510
($H_{0}:r=0,H_{1}:r=1$)	(0.043)	(0.032)	(0.040)	(0.000)	(0.000)	(0.000)
no remaining non-linearity	3.184	0.709	3.199	16.613	1.874	17.022
($H_{0}:r=1,H_{1}:r=2$)	(0.52)	(0.586)	(0.525)	(0.213)	(0.156)	(0.342)
AIC	−6.491			−6.218		
BIC	−6.380			−6.318		

Note: *p* values are in parentheses, and *m* represents the dimension of position parameters.

**Table 3:** The linearity test and no remaining nonlinearity test (transformation variable: *Edu*).

Hypotheses	m=1			m=2		
	$LM_{\chi^{2}}$	$LM_{F}$	LRT	$LM_{\chi^{2}}$	$LM_{F}$	LRT
linearity	15.110	3.574	15.448	27.250	3.303	28.376
($H_{0}:r=0,H_{1}:r=1$)	(0.004)	(0.007)	(0.004)	(0.001)	(0.001)	(0.000)
no remaining non-linearity	2.004	0.444	2.010	10.632	1.194	17.798
($H_{0}:r=1,H_{1}:r=2$)	(0.735)	(0.776)	(0.734)	(0.223)	(0.302)	(0.213)
AIC	−6.429			−6.320		
BIC	−6.318			−6.199		

Note: *p* values are in parentheses, and *m* represents the dimension of position parameters.

**Table 4:** The PSTR model estimation.

Variable	Parameter	(1)		(2)	
ER	$\beta_{01}$	0.2648 **	(2.0725)	0.2382 ***	(3.0611)
Urban	$\beta_{02}$	−8.1218 ***	(−4.4517)	−3.7964 ***	(−6.0713)
Open	$\beta_{03}$	−0.0059 ***	(−4.0843)	−0.4272 ***	(−3.6644)
Edu	$\beta_{04}$	0.1591 ***	(3.4683)	0.1648 ***	(4.9511)
ER	$\beta_{11}$	−0.2054 **	(−1.9865)	−0.1763 ***	(−3.9651)
Urban	$\beta_{12}$	8.2218 ***	(4.3969)	3.9844 ***	(5.9434)
Open	$\beta_{13}$	0.0065 ***	(3.0921)	0.4289 ***	(3.6745)
Edu	$\beta_{14}$	−0.2040 ***	(−4.2733)	−0.1965 ***	(−6.0721)
Slope	γ	6.9560 9.2132		14.0178 7.0954	
Threshold	c				

Note: **, and *** indicate the significance at the 5% and 1% level, respectively. The transformation variable of column 1 is *Pgdp*, and the transformation variable of column 2 is *Edu.*

**Table 5:** The linearity test and no remaining nonlinearity test (transformation variable: *Pgdp*).

Hypotheses	m=1			m=2		
	$LM_{\chi^{2}}$	$LM_{F}$	LRT	$LM_{\chi^{2}}$	$LM_{F}$	LRT
linearity	14.851	3.444	14.891	44.392	5.684	47.489
($H_{0}:r=0,H_{1}:r=1$)	(0.006)	(0.009)	(0.005)	(0.000)	(0.000)	(0.000)
no remaining nonlinearity	5.551	1.246	5.604	7.62	0.848	7.704
($H_{0}:r=1,H_{1}:r=2$)	(0.235)	(0.291)	(0.231)	(0.471)	(0.561)	(0.463)
AIC	−6.408			−6.299		
BIC	−6.297			−6.177		

Note: *p* values are in parentheses, and *m* represents the dimension of position parameters.

**Table 6:** The linearity test and no remaining nonlinearity test (transformation variable: *Edu*).

Hypotheses	m=1			m=2		
	$LM_{\chi^{2}}$	$LM_{F}$	LRT	$LM_{\chi^{2}}$	$LM_{F}$	LRT
linearity	12.945	3.042	13.192	28.487	3.465	29.711
($H_{0}:r=0,H_{1}:r=1$)	(0.012)	(0.018)	(0.001)	(0.000)	(0.000)	(0.000)
no remaining nonlinearity	9.610	2.180	5.271	8.451	0.925	8.251
($H_{0}:r=1,H_{1}:r=2$)	(0.14)	(0.104)	(0.256)	(0.314)	(0.515)	(0.395)
AIC	−6.357			−6.315		
BIC	−6.246			−6.242		

Note: *p* values are in parentheses, and *m* represents the dimension of position parameters.

**Table 7:** Robustness test results.

Variable	Parameter	(1)		(2)	
ER	$\beta_{01}$	0.1319 *	(1.78125)	0.4277 ***	(4.6382)
Urban	$\beta_{02}$	−6.7855 ***	(−5.5466)	−2.6048 ***	(−6.5340)
Open	$\beta_{03}$	−0.0981	(−1.1319)	0.0644 **	(2.0903)
Edu	$\beta_{04}$	0.1634 ***	(5.0133)	0.0282 **	(2.0239)
ER	$\beta_{11}$	−0.0853 ***	(−2.6741)	−0.4073 ***	(−4.6295)
Urban	$\beta_{12}$	6.9612 ***	(5.4562)	2.4943 ***	(6.2262)
Open	$\beta_{13}$	0.0950	(1.1025)	−0.0661 **	(−2.1327)
Edu	$\beta_{14}$	−0.2013 ***	(−6.0926)	−0.0581 ***	(−5.1547)
Slope	γ	7.7591		5.2630	
Threshold	c	8.8453		7.3180	

Note: *, **, and *** indicate the significance at the 10%, 5% and 1% level, respectively. The transformation variable of column 1 is *Pgdp*, and the transformation variable of column 2 is *Edu.*

**Table 8:** The linearity test and no remaining nonlinearity test (transformation variable: *Pgdp*).

Hypotheses	m=1			m=2		
	$LM_{\chi^{2}}$	$LM_{F}$	LRT	$LM_{\chi^{2}}$	$LM_{F}$	LRT
linearity	122.409	42.731	150.851	127.211	22.399	158.339
($H_{0}:r=0,H_{1}:r=1$)	(0.000)	(0.000)	(0.000)	(0.000)	(0.000)	(0.000)
no remaining nonlinearity	2.666	0.593	2.676	8.947	0.999	9.064
($H_{0}:r=1,H_{1}:r=2$)	(0.615)	(0.668)	(0.613)	(0.347)	(0.436)	(0.337)
AIC	−4.065			−4.062		
BIC	−3.955			−3.940		

Note: *p* values are in parentheses, and *m* represents the dimension of position parameters.

**Table 9:** The linearity test and no remaining nonlinearity test (transformation variable: *Edu*).

Hypotheses	m=1			m=2		
	$LM_{\chi^{2}}$	$LM_{F}$	LRT	$LM_{\chi^{2}}$	$LM_{F}$	LRT
linearity	168.926	74.287	231.213	203.391	5.4677	305.603
($H_{0}:r=0,H_{1}:r=1$)	(0.000)	(0.000)	(0.000)	(0.000)	(0.000)	(0.000)
no remaining nonlinearity	4.854	1.06	4.88	5.981	0.662	6.033
($H_{0}:r=1,H_{1}:r=2$)	(0.303)	(0.36)	(0.29)	(0.649)	(0.72)	(0.644)
AIC	−4.598			−4.589		
BIC	−4.487			−4.467		

Note: *p* values are in parentheses, and *m* represents the dimension of position parameters.

**Table 10:** The PSTR model estimation.

Variable	Parameter	(1)		(2)	
ER	$\beta_{01}$	0.0859 **	(2.2932)	0.0540 ***	(6.8134)
Urban	$\beta_{02}$	0.4024 ***	(9.8854)	0.0611 **	(2.1888)
Open	$\beta_{03}$	0.0243 ***	(3.2053)	0.0311 *	(1.8955)
Edu	$\beta_{04}$	−0.0863 ***	(−3.5875)	−0.0232 ***	(−2.7511)
ER	$\beta_{11}$	0.4767 *	(1.8549)	0.4207 *	(1.6959)
Urban	$\beta_{12}$	−0.3035 ***	(−4.1039)	0.5683 ***	(2.8961)
Open	$\beta_{13}$	0.0693	(1.2900)	−0.1366	(−1.6013)
Edu	$\beta_{14}$	0.8545 ***	(5.4040)	0.1608 **	(2.0865)
Slope	γ	2.2403		0.8308	
Threshold	c	10.9898		10.2280	

Note: *, **, and *** indicate the significance at the 10%, 5% and 1% level, respectively. The transformation variable of column 1 is *Pgdp*, and the transformation variable of column 2 is *Edu.*

**Table 11:** The linearity test and no remaining nonlinearity test (transformation variable: *Pgdp*).

Hypotheses	m=1			m=2		
	$LM_{\chi^{2}}$	$LM_{F}$	LRT	$LM_{\chi^{2}}$	$LM_{F}$	LRT
linearity	48.522	12.759	52.257	60.851	8.238	66.886
($H_{0}:r=0,H_{1}:r=1$)	(0.000)	(0.000)	(0.000)	(0.000)	(0.00)	(0.000)
no remaining non-linearity	7.687	1.734	7.773	6.776	1.918	7.194
($H_{0}:r=1,H_{1}:r=2$)	(0.10)	(0.14)	(0.10)	(0.151)	(0.057)	(0.125)
AIC	−3.824			−3.815		
BIC	−3.713			−3.693		

Note: *p* values are in parentheses, and *m* represents the dimension of position parameters.

**Table 12:** The linearity test and no remaining nonlinearity test (transformation variable: *Edu*).

Hypotheses	m=1			m=2		
	$LM_{\chi^{2}}$	$LM_{F}$	LRT	$LM_{\chi^{2}}$	$LM_{F}$	LRT
linearity	119.903	41.396	147.006	134.273	24.421	169.652
($H_{0}:r=0,H_{1}:r=1$)	(0.000)	(0.000)	(0.000)	(0.000)	(0.000)	(0.000)
no remaining nonlinearity	1.617	0.482	1.620	5.853	1.147	3.245
($H_{0}:r=1,H_{1}:r=2$)	(0.656)	(0.695)	(0.655)	(0.213)	(0.165)	(0.225)
AIC	−4.004			−3.987		
BIC	−3.893			−3.875		

Note: *p* values are in parentheses, and *m* represents the dimension of position parameters.

**Table 13:** Robustness test results.

Variable	Parameter	(1)		(2)	
ER	$\beta_{01}$	0.2360 **	(2.2157)	0.8019 **	(2.1036)
Urban	$\beta_{02}$	0.7760 ***	(5.2492)	0.8309	(1.0321)
Open	$\beta_{03}$	0.3530 ***	(6.4596)	1.1748 ***	(3.1661)
Edu	$\beta_{04}$	−0.2954 ***	(−10.9038)	−0.0241 *	(−1.6945)
ER	$\beta_{11}$	0.4425 ***	(6.2127)	0.8421**	(2.1653)
Urban	$\beta_{12}$	−0.8765 *	(−1.9543)	−1.1772 ***	(−3.1992)
Open	$\beta_{13}$	0.3828 ***	(6.8506)	−1.3346	(−0.8818)
Edu	$\beta_{14}$	0.3376 ***	(2.6476)	0.3513 ***	(2.7106)
Slope	γ	5.4533		0.5581	
Threshold	c	9.2153		10.0352	

Note: Note: *, **, and *** indicate the significance at the 10%, 5% and 1% level, respectively. The transformation variable of column 1 is *Pgdp*, and the transformation variable of column 2 is *Edu.*

## Data Availability

The raw/processed data required to reproduce these findings cannot be shared at this time as the data also form part of an ongoing study.
